# Drainage, Irrigation, and Fibrinolytic Therapy (DRIFT) for Adult Intraventricular Hemorrhage Using IRRAflow® Self-Irrigating Catheter

**DOI:** 10.7759/cureus.15167

**Published:** 2021-05-22

**Authors:** Kenan Rajjoub, Ryan M Hess, Timothy E O'Connor, Asham Khan, Adnan H Siddiqui, Elad I Levy

**Affiliations:** 1 Neurosurgery, University at Buffalo Neurosurgery, Buffalo, USA; 2 Neurosurgery, Buffalo General Medical Center, Buffalo, USA; 3 Neurosurgery, Jacobs School of Medicine and Biomedical Sciences, Buffalo, USA

**Keywords:** intraventricular hematoma, aneurysmal subarachnoid haemorrhage, medical device technology development and assessment

## Abstract

Intraventricular hemorrhage (IVH) is a devastating neurosurgical condition associated with high rates of morbidity and mortality. It can occur as the result of several pathologies and typically presents with mental status changes, neurologic deficits, seizures, headaches, and decreased Glasgow Coma Scale score. These patients are often treated with placement of an external ventricular drain, which helps decrease the clot burden; however, they commonly clot off leading to multiple exchanges. We present a case in which drainage, irrigation, and fibrinolytic (DRIFT) therapy using IRRAflow® (IRRAS) irrigating catheter was used to treat a patient with severe IVH secondary to aneurysmal subarachnoid hemorrhage.

## Introduction

Intraventricular hemorrhage (IVH) is a devastating form of intracranial hemorrhage, with an expected mortality rate reported to be between 50% and 80% [1]. The incidence of IVH is high, being reported by Hallevi et al. in their retrospective case series to be present in 45% of the 406 intracranial hemorrhages reviewed in their series. In addition, they reported that patients with IVH are twice as likely to have poor outcome (defined as discharge modified Rankin scale 4 or higher) compared to patients without IVH [2]. IVH typically presents with seizures, headaches, mental status changes, neurologic deficits, and a decreased Glasgow Coma Scale (GCS) score. Placement of an external ventricular drain (EVD) is therapeutic, but ventriculostomy occlusion is a known complication. Fargen et al. reported that 19% of EVDs in their series require at least one replacement and 45% develop at least one occlusion requiring irrigation in order to relieve clot burden [3]. In another study published by Bogdahn et al., 19 out of 100 patients with EVDs developed occlusion of the system. Twelve of these patients carried a diagnosis of subarachnoid hemorrhage [4]. In order to restore patency, flushing with sterile saline is commonly used; however, this often needs to be done multiple times, up to 2.4 irrigations per patient in one series [3]. If flushing fails to restore patency, replacement may be needed. This is associated with an increased risk of infection or new intracranial hemorrhage [3,5].

Self-irrigating catheters such as the IRRAflow® (IRRAS, Stockholm, Sweden) are a relatively new technology. Initial studies have shown benefit when used in the treatment of chronic subdural hematomas (cSDHs). In their limited case series on four patients, Hess et al. reported a length of stay of 4.5 days and a recurrence rate of 0% with combined middle meningeal artery embolization and IRRAflow placement via burr hole [6]. These findings are compared to a median length of stay of three days and a recurrence rate of nearly 30% published in a prior study on patients undergoing operative intervention for cSDH [7]. To our knowledge, no studies describing the use of a self-irrigating catheter, such as the IRRAflow system, for IVH have been published. Given the high rate of failure of standard EVD catheters, we felt that the use of the IRRAflow would be beneficial in the treatment of IVH. Thus, we present our experience using the IRRAflow in the treatment of IVH associated with a ruptured aneurysm.

## Case presentation

**Figure 1:**
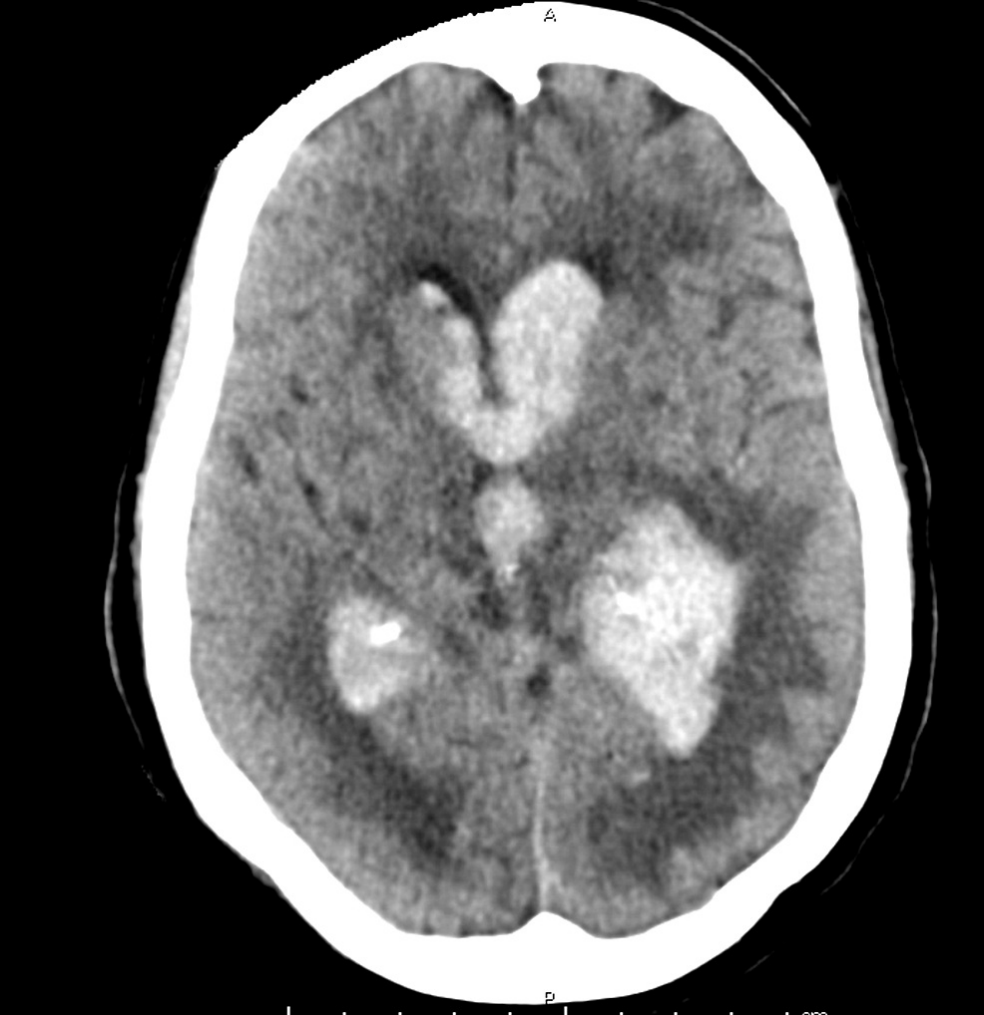
Initial head CT

The patient was a 72-year-old female with a previous medical history of myocardial infarction, hypertension, and type 2 diabetes who presented to the emergency department (ED) after being found unresponsive at home. Per report, she was last known well the previous day when she spoke to her friend. Her friend called her on the day of presentation, and she did not answer. The police were contacted and found her unconscious in her home. On arrival to the ED, the patient had a GCS score of 9. An emergent head computed tomography (CT) was obtained, which showed significant IVH with casted ventricles (Figure 1). CT angiography (CTA) was performed, which showed a left-sided A2-3 junction aneurysm measuring 3.3 x 2.2 millimeters.

**Figure 2:**
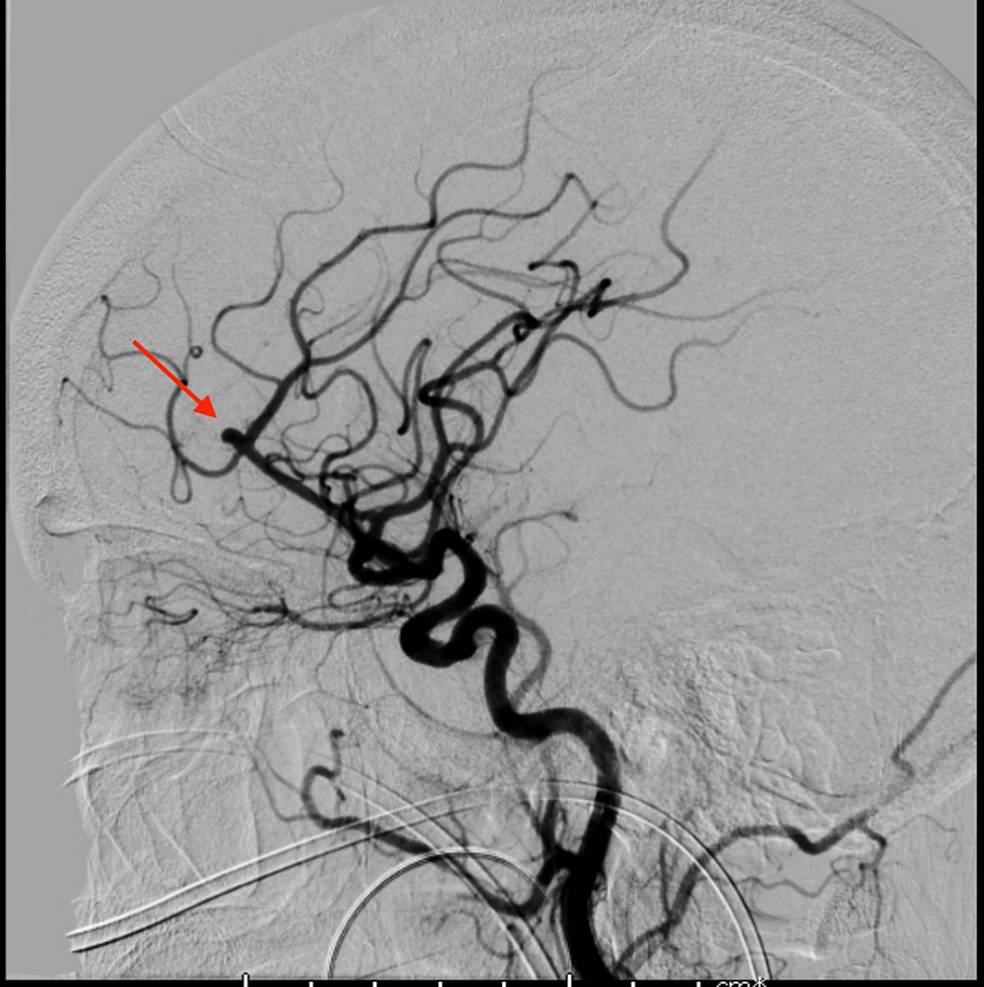
Cerebral angiogram demonstrating the A2-3 junction aneurysm

**Figure 3:**
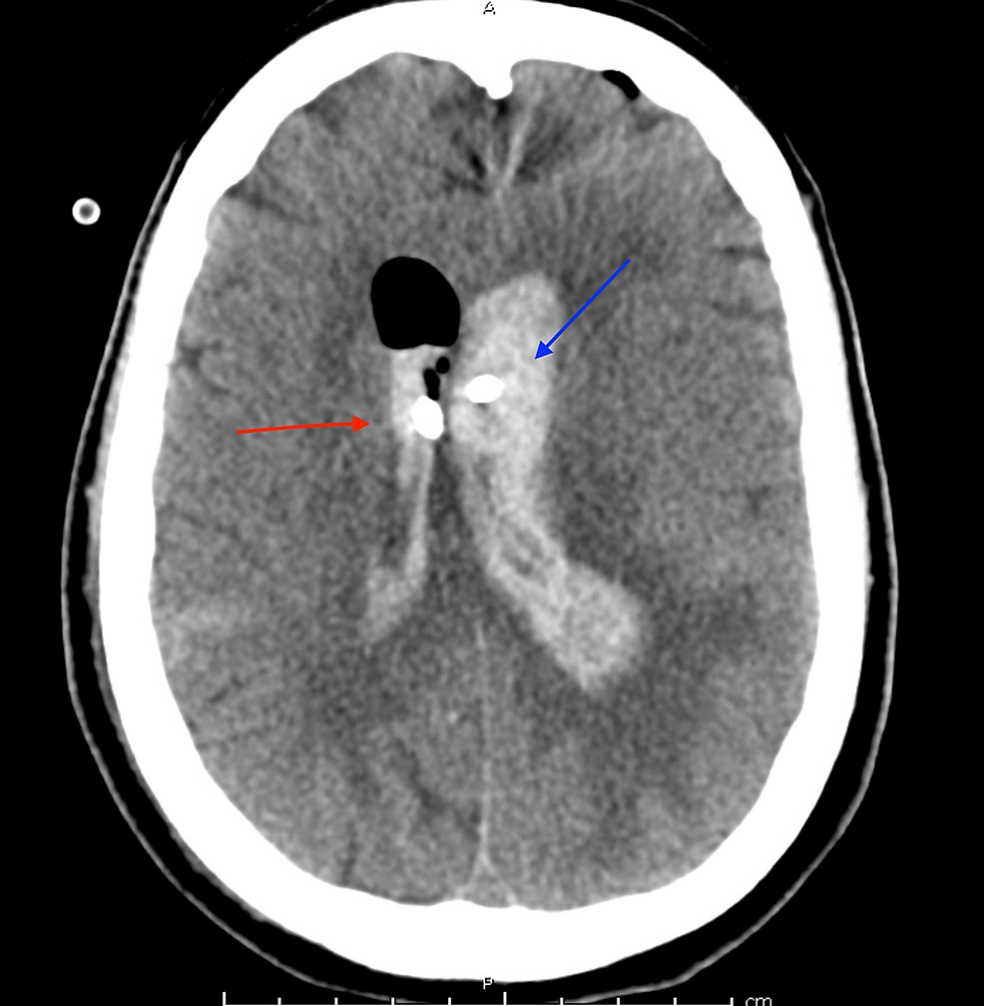
Head CT demonstrating right-sided IRRAflow catheter (red arrow) and left-sided standard catheter (blue arrow)

The patient was emergently transferred to the intensive care unit (ICU) where a Kocher’s point right EVD was placed using the IRRAflow system. The irrigation rate was started at 40 mL/hour. The following morning, the patient was taken for coil embolization of the aneurysm, which went without complication (Figure 2). On hospital day 2, the patient had significant elevation of intracranial pressure (ICP) > 30 mmHg for which a left-sided EVD catheter was placed. Due to supply limitations, a standard EVD catheter was used. Unfortunately, by the following day, the left-sided EVD catheter had become clotted and stopped draining. The decision was made to leave it in place as it was still transducing good ICP waveforms and as current imaging suggested that the ventricular systems were not in communication (Figure 3).

Over the subsequent days of hospitalization, the patient was noted to have multiple examination changes including asymmetry in upper extremity strength and a left gaze deviation. As part of our institutional protocol for aneurysmal subarachnoid hemorrhage, daily transcranial Dopplers are performed. However, this patient had poor temporal windows, making this modality unhelpful. As such, the patient was taken for multiple angiograms, which demonstrated diffuse vasospasm. During each angiogram, verapamil was administered to treat the vasospasm. In addition, continuous electroencephalogram (EEG) was carried out to rule out seizures. No seizures were seen.

**Figure 4:**
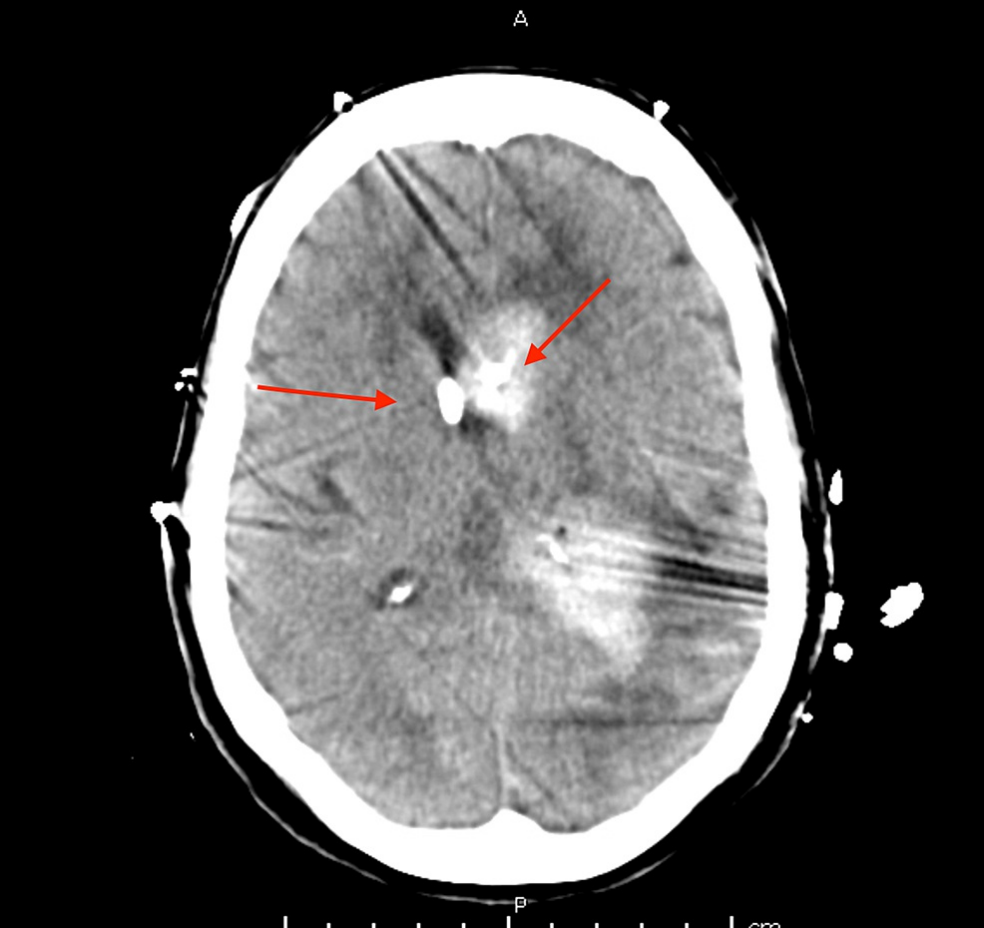
Head CT following placement of left-sided IRRAflow catheter Arrows indicate catheter tips

By hospital day 6, it was clear based upon the head imaging that the ventricle containing the IRRAflow catheter had superior clearance of the hemorrhage (Figure 4). It was decided that a left-sided IRRAflow would be placed to aid ventricular drainage on the left side. Placement went without issue. The following day, intraventricular alteplase (tissue plasminogen activator [tPA]) was started through the right-sided EVD. We followed our typical protocol of clamping of the system for one hour following administration. Irrigation was then continued following the one-hour clamp.

**Figure 5:**
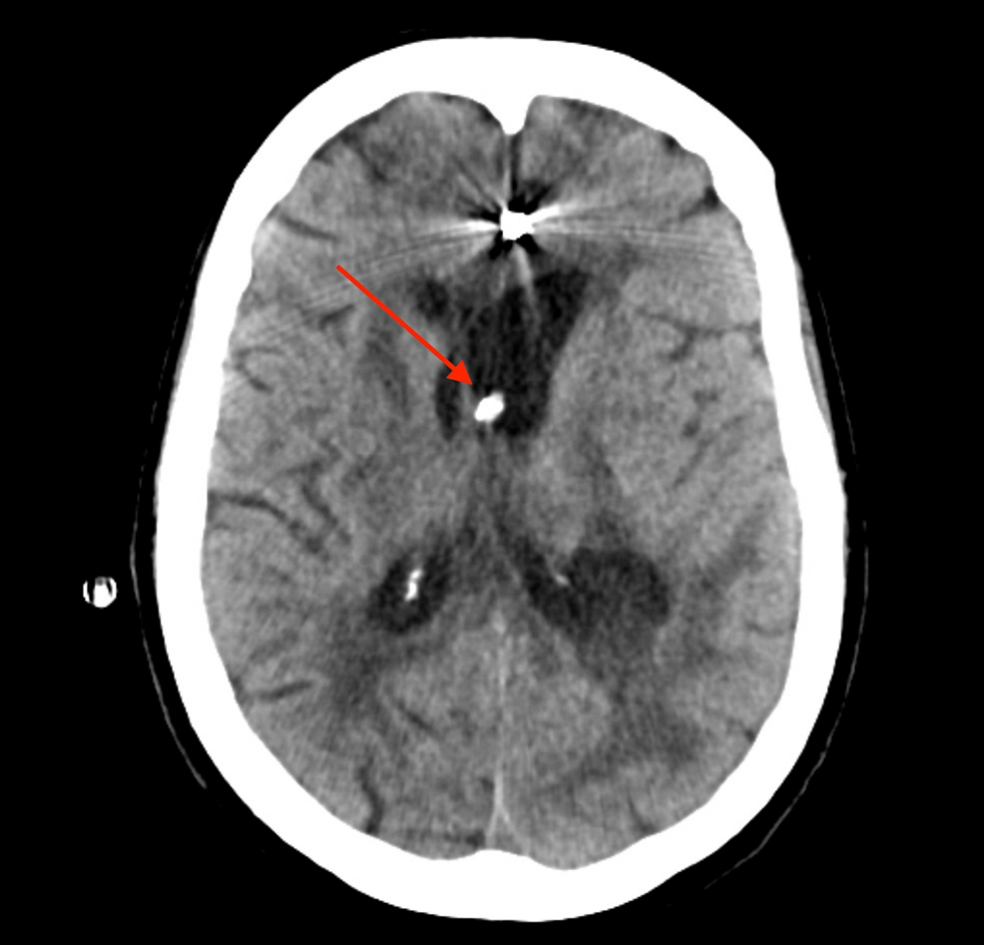
Head CT following removal of right-sided IRRAflow Arrow indicates left IRRAflow catheter tip

Over the subsequent 24-hour period, there was significant drainage through the bilateral EVD systems as the previously clotted left EVD was replaced with a working IRRAflow. The total 24-hour blood-tinged drainage neared 500 mL. Serial images demonstrated a decrease in clot burden and ventricular caliber as a result (Figure 5). From a medical standpoint, the patient was unable to be weaned off the ventilator, and on hospital day 15 the patient underwent tracheostomy and PEG (percutaneous endoscopic gastrostomy) tube placement at the request of the family who still wanted to pursue aggressive care despite the patient’s poor neurological status.

**Figure 6:**
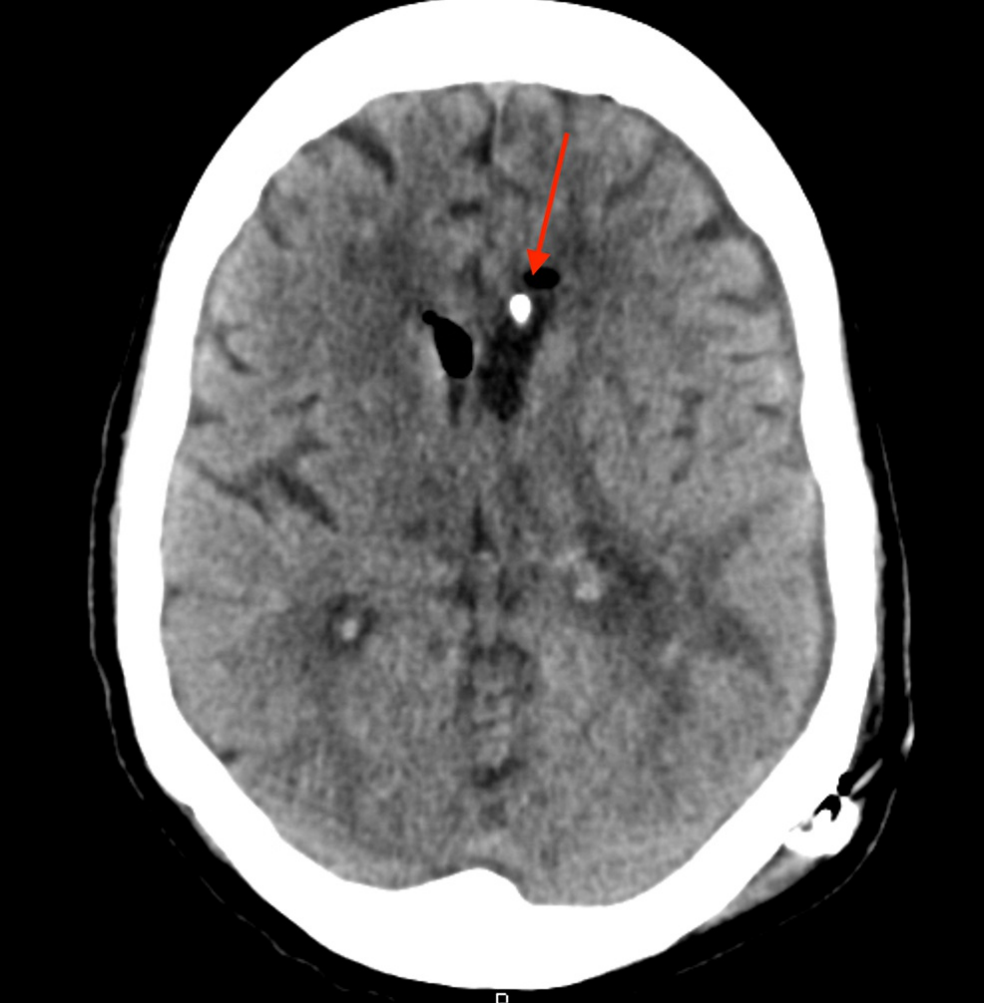
Head CT following ventriculoperitoneal shunt placement Arrow indicates the tip of the shunt catheter

Despite good drainage through the bilateral IRRAflow EVD system, the patient failed several EVD clamp trials to determine shunt dependency. The right-sided EVD was pulled, and the left-sided system was used for testing as this ventricle was persistently enlarged. On hospital day 23, the patient underwent a left ventriculoperitoneal shunt placement given the increased caliber of the left-sided ventricular system. At this time, the ventricular system had been completely cleared of blood (Figure 6).

After shunt placement, the patient was sent to the intermediate level care unit for ventilator weaning. The rest of the patient’s hospitalization was long and complicated by multiple pneumonias and a pulmonary embolus. The neurologic status of the patient never improved, and the patient was minimally responsive despite maximal neurosurgical management. After multiple family discussions, the family elected for comfort measures only and the patient expired after five months of hospitalization.

## Discussion

IVH is often considered a poor prognostic factor in intracranial hemorrhage and is associated with poor neurologic outcome and increased mortality [2]. Of those who survive the initial insult, there is a high rate of shunt dependency due to post-hemorrhagic hydrocephalus (PHH). The pathology of PHH is still not completely clear, though it is believed to arise from a combination of direct occlusion of cerebrospinal fluid (CSF) outflow by small blood clots and scarring of the arachnoid. Data derived from infants with IVH show elevated levels of CSF transforming growth factor beta-1 (TGF-b1) in infants with IVH compared to normal infants. Those infants that later needed shunting showed prolonged elevation of CSF TGF-b1, whereas those that did not need shunt had normalization of this. It is theorized that TGF-b1 leads to an inflammatory cascade, resulting in scarring and obliteration of the normal CSF outflow pathways [8].

Previously described treatment options included ventriculostomy (with or without tPA administration) and endoscopic evacuation. Standard EVD placement is simple and effective; however, it is associated with a high failure rate. Fargen et al. reported a 19-47% rate of catheter failure requiring irrigation in 45% of patients and catheter replacement in 19% of patients. However, these measures can result in new edema or hemorrhage around the catheter, pneumocephalus, and iatrogenic infection [3].

One solution to the high failure rate of EVD is to administer tPA through the catheter in order to aid drainage. The CLEAR III trial examined the impact of intraventricular tPA administration in patients with significant IVH using every eight-hour dosing of 1 mg of tPA for 12 doses when compared to normal saline administration. The trial did not demonstrate a clear benefit of tPA when looking at the number of patients who reached post-hemorrhage modified Rankin score (mRS) of 3 or less. There was also no significant difference in shunt rate between the two groups. However, when examining secondary endpoints, the trial did demonstrate a lower rate of 180-day fatality in patients who had intraventricular tPA without a significant difference in complications when compared to the EVD-only group. In addition, 33% of the tPA group participants achieved 80% or higher clot removal compared to 10% of the EVD-only group [9]. The results of this study seemed to indicate that intraventricular tPA decreased mortality rate and improved drainage through the EVD.

With the increased use of endoscopes to aid in intracranial hemorrhage evacuation, there have been attempts to use this new technology to treat IVH as well. Di Rienzo et al. in their study in 2020 compared the outcome of patients undergoing endoscopic clot evacuation to standard EVD in patients with IVH. Results from their study demonstrated that endoscopic evacuation reduced ICU stay, reduced CSF clearance times, and showed a small mortality benefit. Shunt rates were not significantly different [10].

Even with the above treatment methods, IVH remains a difficult-to-manage pathology without a significant difference in rates of shunt placement. Perhaps the reason for this is that these treatment modalities focus on the removal of blood from the ventricles. Though small blood clots may result in the obstruction of CSF outflow, these methods do little to address the underlying inflammatory process. One novel approach to the treatment of infantile IVH is drainage, irrigation, and fibrinolytic therapy (DRIFT), first implemented in Bristol, United Kingdom. In this approach, temporary frontal and occipital catheters placed under general anesthesia are irrigated with a fibrinolytic and artificial CSF solution until the debris is radiographically cleared. Results published in a 10- year follow-up to the original DRIFT randomized controlled trial showed that infants undergoing this therapy demonstrated significant improvements in cognitive outcome compared to standard treatment consisting of serial lumbar punctures and shunting [11]. This study provides evidence that the inflammatory cascade that occurs after IVH can lead to neurologic morbidity and that continuous irrigation of the CSF can improve outcome due to clearing of pro-inflammatory substances.

To our knowledge, there is little, if any, published data on the use of DRIFT in adults. We present a case report of DRIFT in an adult with IVH using the IRRAflow system. This novel device has been described in the treatment of cSDHs [6], though little data are available on its utility in IVH. Our case demonstrates clear radiographic and clinical superiority of the IRRAflow system compared to standard EVD. The IRRAflow did not need to be replaced and showed excellent irrigation of the patient’s right ventricular system. In fact, the standard catheter was replaced by IRRAflow when the hospital supply was restored. Once intraventricular tPA was administered, there was rapid clearing of blood from the ventricles.

Unfortunately, the patient’s neurological status did not experience significant improvement with our treatment, and shunting was required. Given our institution’s lack of experience using the IRRAflow system for IVH, high rates of irrigation were not used and administration of tPA was delayed by nearly a week. In future cases, we would advocate more aggressive treatment regimens with higher rates of irrigation and early administration of tPA. This, in theory, would promote early clearance of blood degradation products that are thought to lead to a pathologic inflammatory response. It is hard to say if the patient discussed in this case report would have experienced improved outcome with more aggressive management of the IVH. Age likely played a role in outcome as well. Further studies investigating the use of IRRAflow in IVH will need to better elucidate the impact of this variable on outcome, as the infant population studied in the DRIFT trials likely poses improved neuroplasticity.

## Conclusions

The placement of a self-irrigating catheter such as the IRRAflow system is a safe and effective way to avoid complications related to standard EVD placement, such as clotting and replacement. It leads to superior radiographic clearance of hemorrhage from the ventricles and can easily be used as a delivery method for intraventricular tPA to good effect. Theoretically, IRRAflow drainage with tPA could confer similar benefits seen in the DRIFT trial patients, though current data are extremely limited and standardized treatment protocols will be needed to enhance safety and efficacy. Further studies investigating the optimal use of the IRRAflow system are needed to prove its superiority over traditional EVD catheters in the treatment of IVH.
